# Supplementary material for: The Cryptic Bacterial Microproteome
Source: bioRxiv. 2024 Feb 18:2024.02.17.580829. Preprint. [Version 1] doi: 10.1101/2024.02.17.580829 (PMC11188072; doi:10.1101/2024.02.17.580829)
Supplement: Supplement 1 — Figure S1. Venn diagram showing intersection between SmallProt clusters classified as “coding” by RNAcode and EvolScore; ROC curve showing TPR (y-axis) and FPR (x-axis) for the RF model. Related to Figure S2. The hydrophobicity of annotated small proteins and predicted microproteins from Entero67K across all genera Figure S3. The prediction of small proteins homo-oligomers using AlphaFold2 Multimer [file media-1.pdf]

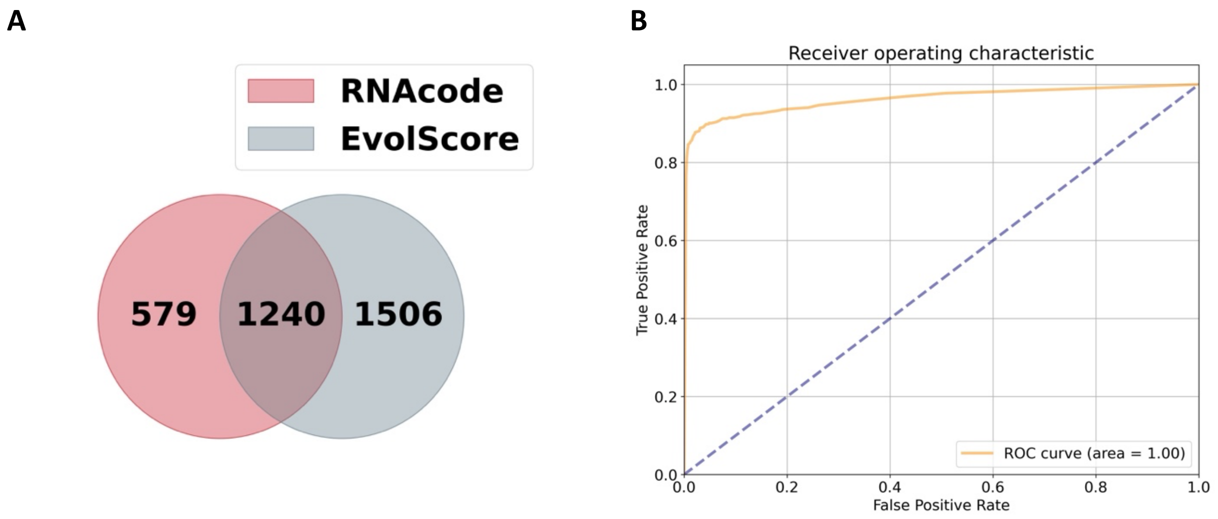

Figure S1. (A) Venn diagram showing intersection between SmallProt clusters classified as "coding" by RNAcode and EvolScore; **(B)** ROC curve showing TPR (*y*-axis) and FPR (*x*-axis) for RF model.

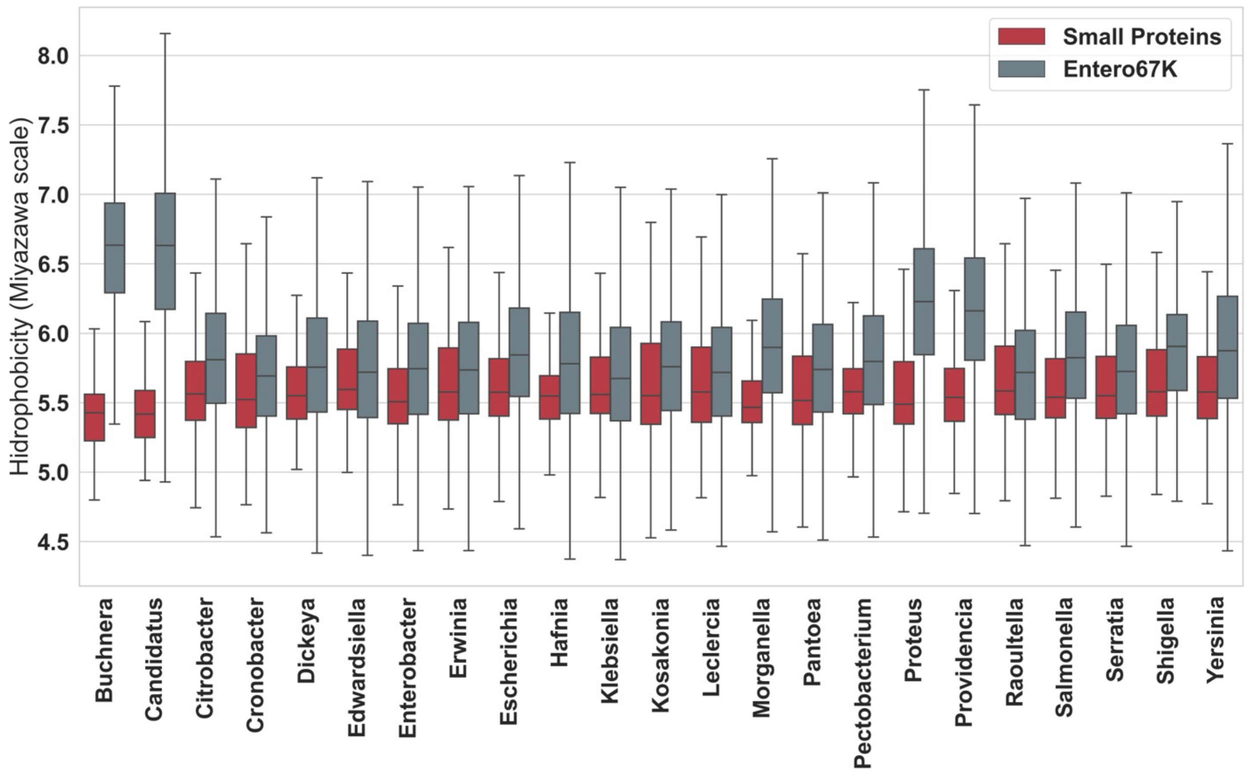

Figure S2. The hydrophobicity of annotated small proteins and predicted microproteins from Entero67K across all genera

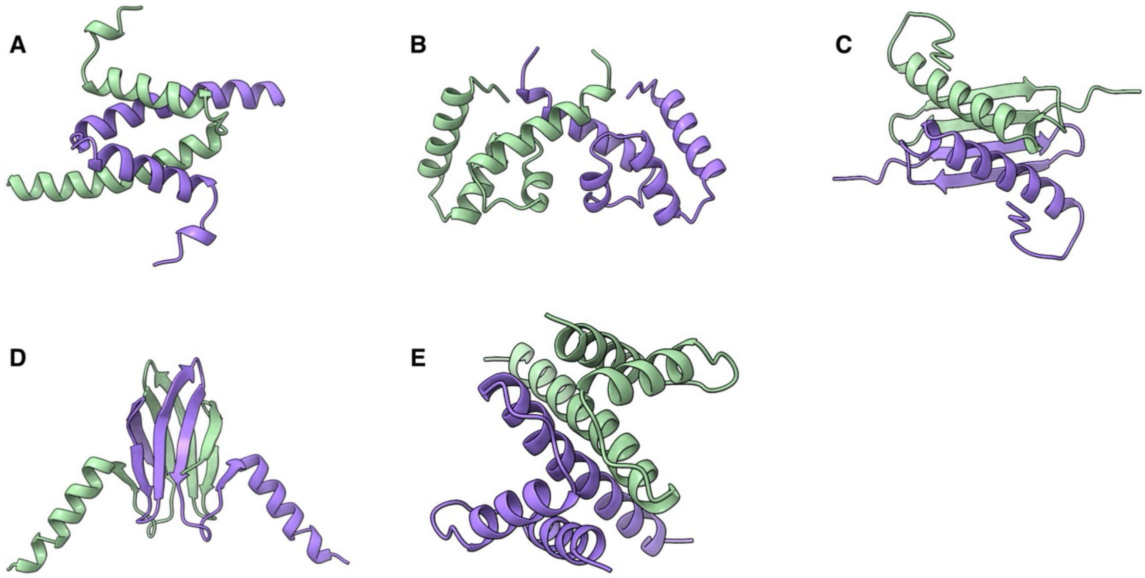

Figure S3. The prediction of small proteins homo-oligomers using AlphaFold2 Multimer
(A) Structure of RsmS protein dimer; **(B)** Structure of Yjb protein dimer; **(C)** Dimeric structure of YoaG proteins; **(D)** Structure of CsrA protein dimer; **(E)** Structure of FumD protein dimer; The chains of the dimers are colored green and purple.
